# Silencing of Plasma Membrane Ca^2+^-ATPase Isoforms 2 and 3 Impairs Energy Metabolism in Differentiating PC12 Cells

**DOI:** 10.1155/2014/735106

**Published:** 2014-09-07

**Authors:** Tomasz Boczek, Malwina Lisek, Bozena Ferenc, Antoni Kowalski, Magdalena Wiktorska, Ludmila Zylinska

**Affiliations:** ^1^Department of Molecular Neurochemistry, Medical University, Mazowiecka 6/8 Street, 92215 Lodz, Poland; ^2^Department of Molecular Biology and Genetics, Science Park, Aarhus University, Gustav Wieds Vej, No. 10, 8000 Aarhus C, Denmark; ^3^Department of Molecular Cell Mechanisms, Medical University, Mazowiecka 6/8 Street, 92-215 Lodz, Poland

## Abstract

A close link between Ca^2+^, ATP level, and neurogenesis is apparent; however, the molecular mechanisms of this relationship have not been completely elucidated. Transient elevations of cytosolic Ca^2+^ may boost ATP synthesis, but ATP is also consumed by ion pumps to maintain a low Ca^2+^ in cytosol. In differentiation process plasma membrane Ca^2+^ ATPase (PMCA) is considered as one of the major players for Ca^2+^ homeostasis. From four PMCA isoforms, the fastest PMCA2 and PMCA3 are expressed predominantly in excitable cells. In the present study we assessed whether PMCA isoform composition may affect energy balance in differentiating PC12 cells. We found that PMCA2-downregulated cells showed higher basal O_2_ consumption, lower NAD(P)H level, and increased activity of ETC. These changes associated with higher [Ca^2+^]_c_ resulted in elevated ATP level. Since PMCA2-reduced cells demonstrated greatest sensitivity to ETC inhibition, we suppose that the main source of energy for PMCA isoforms 1, 3, and 4 was oxidative phosphorylation. Contrary, cells with unchanged PMCA2 expression exhibited prevalence of glycolysis in ATP generation. Our results with PMCA2- or PMCA3-downregulated lines provide an evidence of a novel role of PMCA isoforms in regulation of bioenergetic pathways, and mitochondrial activity and maintenance of ATP level during PC12 cells differentiation.

## 1. Introduction

Neuronal development is highly organized sequence of events eventually leading to the formation of functional nerve cells. Neurogenesis occurs globally during development and, to some extent, is also active in adult nervous system [1]. The formation of new neurites is critically dependent on the adequate energy supply. However, the degree to which glycolysis or oxidative phosphorylation (OxPh) contributes to energy provision remains controversial. It is now a textbook fact that complete glucose oxidation is the most energetically favorable and provides nearly 87% of total ATP [2, 3]. Because of high ATP yield during OxPh (26 of 30 ATP molecules are harvested this way), one may assume that during development ATP will also be synthesized mostly by mitochondria. This is supported by studies in primarily cultures isolated from embryos and postnatal cultured neurons, both showing ATP derived from OxPh [4]. Mitochondrial prevalence in ATP synthesis during differentiation could be also highlighted by a large number of mitochondria distributed throughout the length of axons and in presynaptic terminals [5].

Ca^2+^, as a link between ATP handling and neurogenesis is suggested by numerous studies [6, 7]. Transient elevations of cytosolic Ca^2+^ concentration ([Ca^2+^]_c_) may on the one hand boost ATP synthesis but, on the other, ATP is required for homeostatic maintenance of a low resting [Ca^2+^]_c_ in neuronal cells [8]. In the differentiation process plasma membrane Ca^2+^ ATPase (PMCA), an ATP-consuming pump extruding cytosolic Ca^2+^, is considered as one of the major players for Ca^2+^ homeostasis. PMCA exists in four isoforms PMCA1–4, which differ considerably by basal activity, Ca^2+^ affinity, and tissue distribution [8]. The fastest PMCA2 and PMCA3 isoforms are expressed predominantly in excitable cells and are termed neurospecific. PMCA1 and PMCA4 are abundantly expressed and perform a housekeeping function. The expression of particular PMCAs is developmentally regulated, what has been shown at mRNA and protein level [9–11]. It is believed that changes in the expression of particular variants of the pump are spatially and temporary controlled to regulate the magnitude and duration of Ca^2+^ signals during differentiation. Recently, it has been demonstrated that PMCA acting as Ca^2+^/H^+^ countertransporter is also a major source of cellular protons [12]. In view of this finding and taking into consideration the different kinetic parameters, PMCA isoforms may be an important regulator of cellular bioenergetics and ATP demands during differentiation.

To evaluate this, we used differentiated PC12 cells obtained after transfection with eukaryotic vectors containing antisense sequences designed to either PMCA2 or PMCA3. The PC12 lines with stable downregulated expression of PMCA2 or PMCA3 were validated in our several other studies [13–15] showing the reduction of PMCA2 or PMCA3 protein level by almost 50%. PC12 cells with their unique features characteristic for sympathetic-like neurons are a well-established model for studying the processes occurring during neurite outgrowth. Additionally, upon induction of differentiation they not only become more neuronal in the sense of oxidative metabolism predominance but also rely on glycolysis for ATP supply [16]. Using stable transfected PC12 lines we could control the level of PMCA isoforms and monitor long-time effects of their suppression. Therefore, we have attempted to answer whether neuron-specific PMCA isoforms may affect energy balance in differentiating cells and whether their presence confers a survival advantage during energy deprivation.

## 2. Materials and Methods

### 2.1. Reagents

Reagents, if not otherwise stated, were purchased from Sigma-Aldrich (Germany). The PC12 rat pheochromocytoma cell line was obtained from ATCC (USA) or Sigma-Aldrich (Germany). RPMI 1640 medium was from PAA (Austria). Calf and horse sera were from BioChrom (UK). Annexin V-FITC Apoptosis Detection Kit was purchased in Roche Diagn. (Germany). Alexa Fluor 488 and Fluo-4 Calcium Assay kit were from Life Technologies (USA). Protein Assay Kit was from Bio-Rad (USA). Primary antibodies against *β*III-tubulin and GAPDH were from Santa Cruz Biotech. (USA).

### 2.2. Cell Culture and Differentiation

PC12 rat pheochromocytoma cells were routinely maintained in RPMI-1640 medium containing 10% horse serum, 5% fetal bovine serum, 25 mM HEPES, pH 7.4 (21°C), 2 mM L-glutamine, 25 U/mL penicillin, and 25 *μ*g/mL streptomycin in a humidified incubator at 37°C with 5% CO_2_. Nearly 50% reduction in PMCA2 or PMCA3 protein level was achieved using an antisense RNA cloned into pcDNA3.1(+) vector transfected to naive PC12 cells. Following selection with increasing G418 concentration (up to 1 mg/mL), we obtained stably transfected lines, in which PMCA2 (_2 line) or PMCA3 (_3 line) was downregulated. PC12 cells carrying an empty vector were used as a control (C). Plasmids construction, clones selection, and characterization were described previously [14]. Differentiation process was induced with 1 mM dibutyryl-cAMP (db-cAMP). The cells were cultured in the presence of differentiating agent for another 48 h and all the results presented here were obtained following 2-day differentiation process. Routinely, no more than 12 passages were used and the expression level of PMCA2 and PMCA3 was controlled every 4 passages. Because PC12 cells exhibit some level of variability, we separately transfected two PC12 lines of different sources to increase fidelity and maintain the reproducibility of our results.

### 2.3. Microscopic Analysis

Cell morphology was analyzed with an Olympus CK-40 inverted microscope and images were captured using a CCD camera. For confocal imaging, ~10^3^ cells seeded on poly-L-lysine coated glass LabTek II chamber slides were fixed with 3.8% paraformaldehyde for 30 min at room temperature, permeabilized with 0.1% Triton X-100 for 10 min at 4°C, and blocked with 6% BSA for 3 h at room temperature. Fixed cells were then overnight incubated with mouse monoclonal anti-*β*III-tubulin (1 : 150) at 4°C followed by incubation with secondary anti-mouse antibodies conjugated to Alexa Fluor 488 (1 : 1000) for 2 h at room temperature. Images were taken on TCS SP5 confocal laser scanning microscope with 63x objective (Leica). The average fluorescence intensity after background subtraction was measured with Leica LAS AF Lite software (Leica). Mitochondrial mass was quantified with MitoTracker Green FM. In this method, differentiated cells were first loaded with 150 nM MitoTracker Green FM for 30 min at 37°C and then fixed and imaged as described above.

For electron microscopy, cells were fixed with 3% paraformaldehyde and 1% glutaraldehyde in 100 mM phosphate buffer for 1 h at room temperature. Then, they were dehydrated in increasing ethanol concentration: 25% for 5 min, 50% for 10 min, 75% for 15 min, 90% for 20 min, and 99.8% for 2 × 20 min. Following dehydration, cells were infiltrated in LR White resin : 99.8% ethanol (1 : 1) for 30 min at room temperature. The embedded cells were encapsulated in pure LR White resin for solidification. The process was carried out for 12 h at 37°C, 12 h at 46°C, and 48 h at 56°C. The ultrathin sections were transferred to carbon-sprayed nickel grids, counterstained with 2.5% uranyl acetate and lead citrate for 30 min, and observed with JEOL JEM 1010 transmission electron microscope. Mitochondria-to-cell-volume ratio was calculated by Cavaileri estimator using Stereo-Investigator (MBF Bioscience).

### 2.4. Ca^2+^ Measurement

Approximately 1 × 10^4^ differentiated cells in each well of 96-well plate were loaded with 10 *μ*M Fluo-4 for 1 h at 37°C. The fluorescence monitored in a kinetic mode on Victor X3 plate fluorometer was recorded using 488 nm excitation filter and 535 emission filter. Appropriate controls for estimation of background fluorescence including phenol-red-free RPMI medium, Fluo-4 solution alone, and cell-free recording solution were included. Changes in Fluo-4 fluorescence were converted to absolute [Ca^2+^]_c_ according to the equation [Ca^2+^]_free_ = *K*_*d*_((*F* − *F*_min⁡_)/(*F*_max⁡_ − *F*)), where *K*_*d*_ = 345 nM. Maximal signal (*F*_max⁡_) was obtained with 10 *μ*M ionomycin, while the minimal signal (*F*_min⁡_) was obtained with 10 mM EGTA. In a separate set of experiments, cells were preincubated for 30 min with 10 *μ*M BAPTA-AM and [Ca^2+^]_c_ were monitored as above.

### 2.5. Drug Treatment

If not stated otherwise, the given parameters were assessed in a buffer containing 5 mM D-glucose (+glucose). 1 mM KCN was added alone 20 min before measurement to glucose-containing buffer (+glucose + KCN). The contribution of glycolysis to ATP synthesis was determined with 2-DG (20 *μ*M) added in presence of glucose and pyruvate (5 mM and 1 mM, resp.) 2 h before measurement (+glucose + 2-DG + pyruvate). Short (3 h treatment) and long (48 h treatment) time effects of 6 *μ*M oligomycin action were determined in glucose-containing buffer.

### 2.6. Flow Cytometry Analysis

Cells were incubated with either 6 *μ*M oligomycin (for 3 h or 48 h) or with 20 *μ*M 2-deoxyglucose (for 2 h) in the presence of glucose. ~1 × 10^6^ cells were double stained with Annexin V/Propidium iodide using Annexin V-FITC Apoptosis Detection Kit I according to the manufacturer's protocol and analyzed with FACScan Becton Dickinson. The fluorescence recorded from 10^4^ cells was measured in each experiment. The data were plotted using CellQuest Becton Dickinson software. The basal level of mortality was determined with or without 0.1% DMSO (solvent for oligomycin) in the presence of glucose. Due to lack of differences, the data obtained without DMSO were chosen for further comparisons.

### 2.7. ATP Measurement

ATP was measured in nontreated cells and upon treatment with inhibitors, as described in Drug Treatment section. ATP concentration was determined using adenosine 5′-triphosphate (ATP) Bioluminescent Assay Kit on GloMax 20/20 luminometer (Promega) and normalized to the protein content. In experiments with oligomycin, cells incubated with 0.1% DMSO (oligomycin solvent) were used as a negative control and ATP values from these cells were subtracted from ATP values obtained in oligomycin (+) experiments. For each set of measurements a second negative control (no cells) was included, and a background fluorescence was further subtracted from all other values.

### 2.8. Glucose Consumption and Lactate Release

Medium of a 48 h culture of differentiated cells was used to quantify basal glucose consumption and lactate release using Lactate Assay Kit and Glucose Assay Kit. The same method was used to assessed glucose and lactate concentration following treatments with inhibitors (see Results and Drug Treatment sections).

### 2.9. Enzymatic Activities

For citrate synthase activity, ~1 × 10^6^ cells were suspended in a buffer containing 50 mM TRIS-HCl, pH 8.0, 50 mM acetyl-CoA, 0.1% Triton X-100, and 100 mM dithionitrobenzoic acid (DTNB) and the reaction was initiated by the addition of oxaloacetate to a final concentration of 250 mM. After 5 min of incubation the absorbance of a resulting product thionitrobenzoic acid (TNB) was determined spectrophotometrically at 412 nm [17]. The activity of ETC complexes I–III was assessed at 37°C in mitochondrial fraction obtained as described in [18] using Beckman DU 640 spectrophotometer and are expressed as nmol/min after normalization to citrate synthase activity. The activity of complex I was measured as a rate of NADH oxidation using decylubiquinone as an electron acceptor. The absorbance was monitored at 340 nm with 380 nm as a reference wavelength and the rate sensitive to rotenone (10 *μ*M) was taken as complex I activity [19]. Complex II activity was measured by the reduction of 2,6-dichlorophenolindophenol (DCIP). The reaction was initiated by the addition of 50 *μ*M decylubiquinone in the presence of succinate, KCN, and rotenone. The activity of complex II was calculated based on the rate of DCIP reduction at 600 nm with a reference wavelength of 520 nm [20]. Complex III activity was measured by monitoring of cytochrome c reduction at 550 nm. The 580 nm wavelength was used as a reference. In parallel experiment 50 *μ*M rotenone was added to the incubation buffer to assess rotenone-insensitive activity [21]. Complex IV activity was determined in digitonin-permeabilized cells prepared as described in [22], using an oxygraph (Anton Paar). ~2 × 10^6^ permeabilized cells suspended in a reaction buffer containing 50 mM MOPS, pH 7.5, 0.3% Tween 20, and 1 mM FCCP were injected into polarographic chamber. Subsequently, 10 mM ascorbate and 0.3 mM tetramethyl-p-phenylenediamine (TMPD) were added and the oxygen consumption was recorded for 5 min. Then, 700 *μ*M KCN was added and the respiration was measured for additional 5 min. The complex IV activity obtained by subtracting the KCN-insensitive respiration was calculated by an accompanying software and is expressed as nmol O_2_/min after normalization to citrate synthase activity.

### 2.10. Monitoring of NAD(P)H and Oxygen Uptake

NAD(P)H autofluorescence was measured at 30°C in a kinetic mode on Victor X3 multilabel plate fluorometer using an excitation wavelength of 350 nm with emission recorded at 450 nm. 1 × 10^4^ cells were seeded per well of 96-well plate and differentiated for 48 h. Then, the culture medium was changed to serum-free RPMI containing 5 mM glucose and after stabilization of the signal, 2 mM KCN was applied to obtain a maximal fluorescence (positive control), and 1 *μ*M FCCP was used to monitor a minimal signal (negative control).

The endogenous respiratory rate was measured at 37°C using OROBOROS oxygraph (Anton Paar) with a computer-interfaced Clark-type electrode. ~1 × 10^7^ cells/mL suspended in a buffer containing 10 mM HEPES, pH 7.4, 250 mM sucrose, 1% BSA, and 1 mM potassium phosphate were allowed to reach O_2_ and temperature equilibrium for 3 min to record a basal signal. Respiration was inhibited by the addition of 2 mM KCN and the minimal O_2_ consumption was monitored for 2.5 min. For uncoupled respiration measurements, 1 *μ*M FCCP was added and the maximal O_2_ consumption was further monitored for 3 min. Respiratory rate was expressed as nmol O_2_ consumed/mg/min.

### 2.11. Real-Time PCR

Total cellular RNA was extracted using Trizol reagent according to the manufacturer's protocol. cDNA was synthesized using 1 *μ*g of isolated RNA, oligo(dT) primers, and M-MLV reverse transcriptase in a 20 *μ*L of reaction mixture. PMCA2 or PMCA3 gene expression level was quantified using SYBR Green fluorescent dye in the following conditions: 15 min at 95°C followed by 40 cycles at 95°C for 15 s, 60°C for 30 s, and 72°C for 30 s. PCR reactions were performed in an AbiPrism 7000 sequence detection system (Applied Biosciences). The relative fold change after normalization to Gapdh expression was calculated using a comparative 2^−ΔΔCt^ method [23].

### 2.12. Western Blot

40 *μ*g of total cell lysate prepared as described in [15] was resolved on a 10% SDS-PAGE gel and electroblotted onto nitrocellulose membranes. Membranes were blocked with 5% BSA in TBS-T buffer (10 mM TRIS-HCl, pH 7.4, 150 mM NaCl, and 0.05% Tween-20) for 1 h at room temperature and incubated overnight at 4°C with either anti-PMCA2 (1 : 1000), anti-PMCA3 (1 : 1000), anti-*β*-actin (1 : 1000), anti-GAPDH (1 : 2500), or anti-*β*-III tubulin (1 : 1000) antibodies followed by 4 h incubation with secondary antibodies (1 : 10000) coupled to alkaline phosphatase. BCIP/NBT was used according to the manufacturer's instructions to visualize immunoreactive bands. Blots were scanned and quantified using GelDocTMEQ system with Quantity One 1-D Analysis Software version 4.4.1 (Bio-Rad).

### 2.13. Statistical Analysis

The data are shown as means ± SEM of *n* separate experiments (*n* ≥ 3). Statistical analyses were done using STATISTICA 8.0 (StatSoft) with ANOVA test and *P* value <0.05 was considered as statistically significant.

## 3. Results

### 3.1. PMCA2- or PMCA3-Downregulated Differentiated PC12 Lines

**Figure 1:**
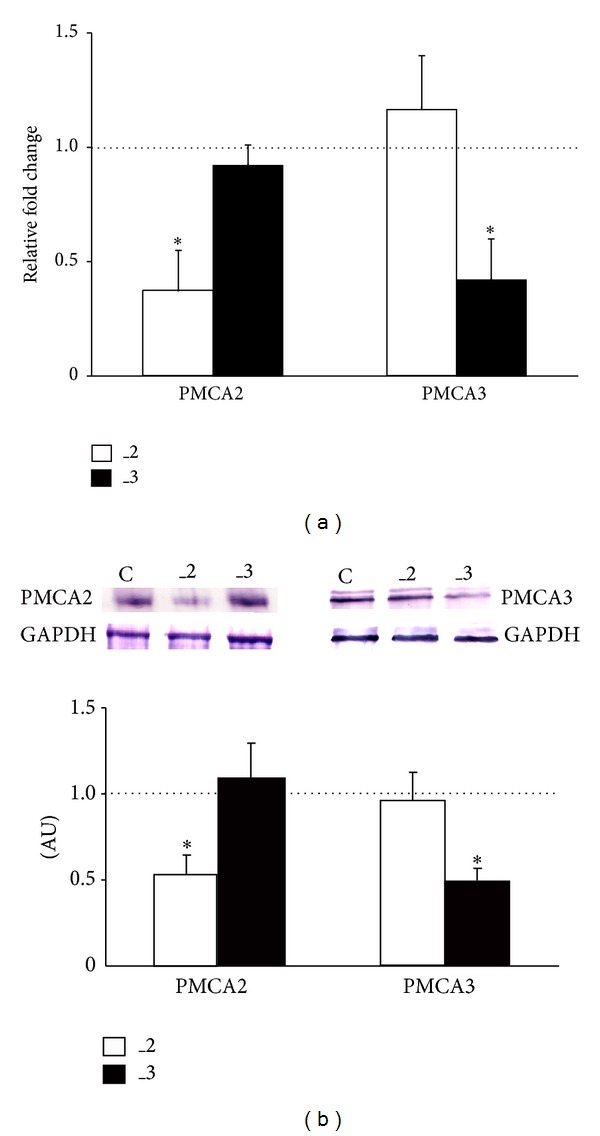
Efficiency of PMCA isoforms downregulation in differentiated PC12 cells. (a) The relative amount of PMCA2 or PMCA3 transcripts was evaluated by real-time PCR with Gapdh used as a reference gene. The relative fold change was calculated using 2^−ΔΔCt^ method. The level of target gene expression in control line was taken as 1 (dotted line). *P < 0.05, PMCA-deficient lines versus control cells. (b) Western blot analysis of PMCA2 and PMCA3 protein level. The results are shown as arbitrary units (AU) obtained after normalization to endogenous GAPDH content. The level of either PMCA2 or PMCA3 protein in control line was taken as 1 (dotted line). *P < 0.05, PMCA-deficient lines versus control cells. C: mock-transfected PC12, _2: PMCA2-reduced PC12, and _3: PMCA3-reduced PC12.

Real-time PCR analysis revealed that experimental downregulation of PMCA2 or PMCA3 in differentiated PC12 cells [15] significantly decreased transcript level by 63 ± 13% and 58 ± 15% for PMCA2 and PMCA3, respectively (Figure 1(a)). Furthermore, changes in mRNA content corresponded to an approximate ~50% reduction in PMCA isoforms protein amount (Figure 1(b)).

**Figure 2:**
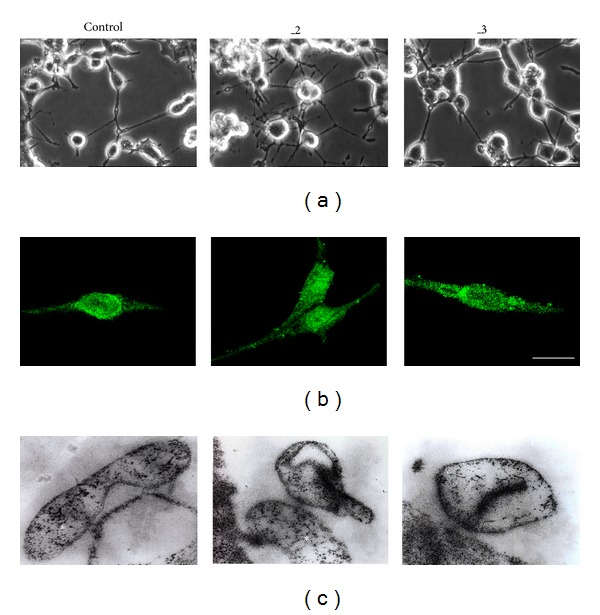
Microscopic characteristic of differentiated PC12 lines. (a) Morphological changes induced by differentiation with 1 mM db-cAMP. The morphology was analyzed with an Olympus CK-40 inverted microscope and images were captured using a CCD camera. Scale bar 10 *μ*m. (b) Evaluation of mitochondrial mass with MitoTracker Green TM in fixed cells using TCS S5 confocal microscope. Scale bar 20 *μ*m. (c) Representative micrographs of cell ultrastructure at 105.000x magnification with mitochondria marked with asterisks. Control: mock-transfected PC12, _2: PMCA2-reduced PC12, and _3: PMCA3-reduced PC12.

Additionally, our previous study revealed that 48 h treatment with db-cAMP accelerated differentiation process of PC12 lines [15]. In comparison to the mock-transfected control cells, PMCA-deficient lines exhibited more intensive formation of neurite network, particularly visible in PMCA2-downregulated line (Figure 2(a)). To analyze the bioenergetic processes, we first assessed the number of mitochondria in examined lines using MitoTracker Green TM (Figure 2(b)). Single-cell fluorescence level indicated a similar mitochondrial mass in all lines (365 ± 24, 382 ± 38, and 344 ± 19 units of fluorescence intensity in Control, _2, and _3 lines, resp.). Also, despite the different shape of mitochondria visualized in ultrastructural studies (Figure 2(c)), the average mitochondria-to-cell-volume ratio calculated using Stereo-Investigator was comparable between lines (0.056 ± 0.008; 0.0515 ± 0.004; and 0.0555 ± 0.011 for C, _2, and _3, resp.). Given that activity of citrate synthase is considered as biochemical determination of mitochondrial mass, we next assayed the enzyme activity, which was 432 ± 21, 499 ± 28, and 428 ± 16 nmol/mg/min for C, _2, and _3, respectively. It confirmed, in addition to microscopic analysis, a similar level of functional mitochondria in all examined cell lines.

As we have already reported [15], PMCA2 or PMCA3 knockdown resulted in permanent increase in resting [Ca^2+^]_c_ and these changes were associated with reduced protein level of examined PMCA isoforms. Thereby, we next clarified the possible relationship between [Ca^2+^]_c_ and ATP content (Table 1). A short-time (30 min) treatment with calcium chelator BAPTA-AM decreased [Ca^2+^]_c_ in PMCA-reduced lines, but only in _2 line that this change correlated with diminished ATP level. It suggests a close link between Ca^2+^ and ATP levels in PMCA2-reduced cells.

### 3.2. Contribution of Glycolysis versus Oxidative Phosphorylation to Cellular ATP Maintenance

**Figure 3:**
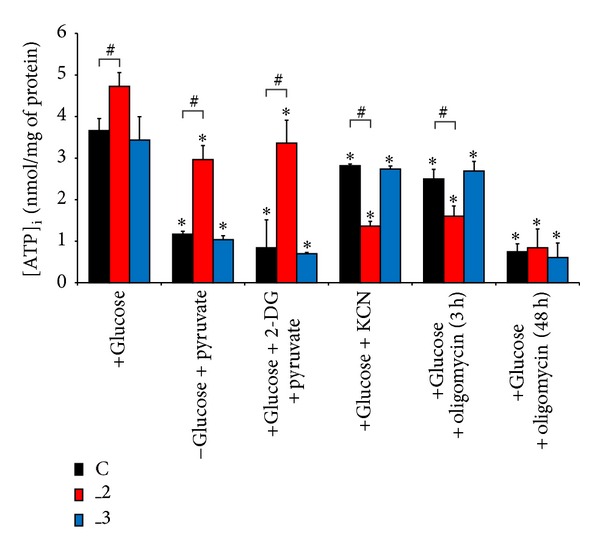
ATP amount in differentiated PC12 lines after drug treatment. ATP level was quantified luminometrically and calculated using ATP standard and normalized to the protein content in the sample. The results are averages of *n* = 7 experiments performed in duplicate, using different cell cultures. *P < 0.05 versus control untreated cells, ^#^ *P* < 0.05 treated versus untreated cells. C: mock-transfected PC12, _2: PMCA2-reduced PC12, and _3: PMCA3-reduced PC12.

Next we assessed the effect of PMCA isoforms downregulation on glycolysis and OxPh contribution to ATP supply in differentiated PC12 lines (Figure 3). Under steady state conditions (with glucose in medium) the highest intracellular ATP level was detected in _2 line, and it was ~30% higher than that of control, mock-transfected cells. In connection with microscopic analysis this change is unlikely to result from increased mitochondrial volume or mitochondrial mass and simply indicates higher ATP concentration in individual cell.

Cultivation of cells for 15 min in glucose-free medium, but with addition of pyruvate, decreased ATP content in all lines, more intensively in control and _3 lines. It is noteworthy that observed ATP values reflected mainly the effectiveness of OxPh. To evaluate contribution of glycolysis to ATP synthesis we added 2-DG in the presence of glucose and pyruvate to inhibit glycolytic hexokinase. Following 2 h treatment, ATP content in all lines was reduced to the level previously detected in glucose-free, but pyruvate supplemented conditions. Treatment with cyanide for 20 min to block mitochondrial ETC decreased ATP by 23%, 71%, and 20% in C, _2, and _3 lines, respectively. Similar results were obtained after incubation for 3 h in the presence of oligomycin. This indicates that PMCA2 preferentially utilizes glycolytic pathway for ATP production, whereas remaining PMCA isoforms rely more on the OxPh. Moreover, culturing cells in the presence of oligomycin for 2 days revealed a dramatic reduction in ATP content in all lines, also showing a prevalence of OxPh in energy generation over a long period of time.

### 3.3. Energy Deprivation Induced Cell Death in Differentiated PC12 Cells

**Figure 4:**
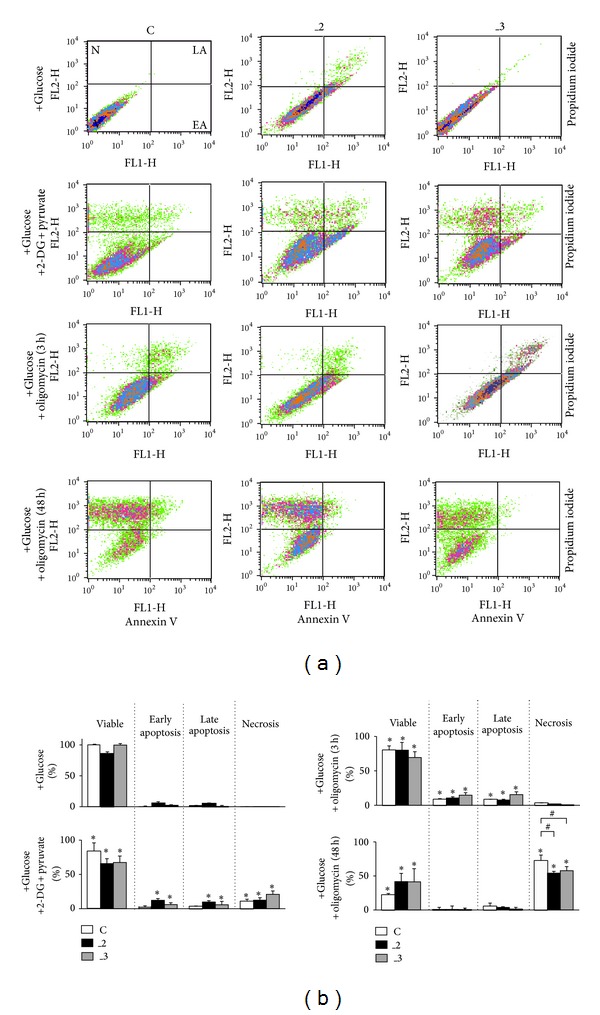
Flow cytometry analysis of differentiated PC12 cells after drug treatment. (a) The analysis was performed using Annexin V-FITC/propidium iodide staining (FACScan, Becton Dickinson). Typical dot plots are presented (*n* = 3 independent experiments with similar results) and indicate N: necrotic cells, V: live cells, LA: late apoptosis, and EA: early apoptosis. (b) Quantification of apoptosis and necrosis after drug treatment. The bars indicate the distribution (% ± SD) within cell population. Data shown are the mean of *n* = 3 independent experiments. Quantification of cells was done using CellQuest software (Becton Dickinson). *P < 0.05 versus corresponding untreated cells; ^#^ *P* < 0.05 versus treated control line as indicated. C: mock-transfected PC12, _2: PMCA2-reduced PC12, and _3: PMCA3-reduced PC12.

To resolve possible consequences of energy deprivation on cell survival, a double staining with Annexin V and propidium iodide was done (Figure 4). It showed that inhibition of hexokinase, even in the presence of pyruvate, increased necrotic cell number in all lines, as well slightly enhanced apoptosis in _2 and _3 cells. Treatment with oligomycin for 3 h reduced cell viability in all lines by increasing apoptosis (but not necrosis). These results suggest that neither glycolysis alone nor OxPh are able to sufficiently protect cells against death. A massive necrosis was induced by oligomycin after 48 h which was, however, less severe in _2 and _3 lines. Due to highly deleterious effect of prolonged oligomycin treatment, in further experiments we decided to limit its action to 3 h.

### 3.4. PMCA2- and PMCA3-Deficient Cells Exhibit Higher Glycolytic Activity

**Figure 5:**
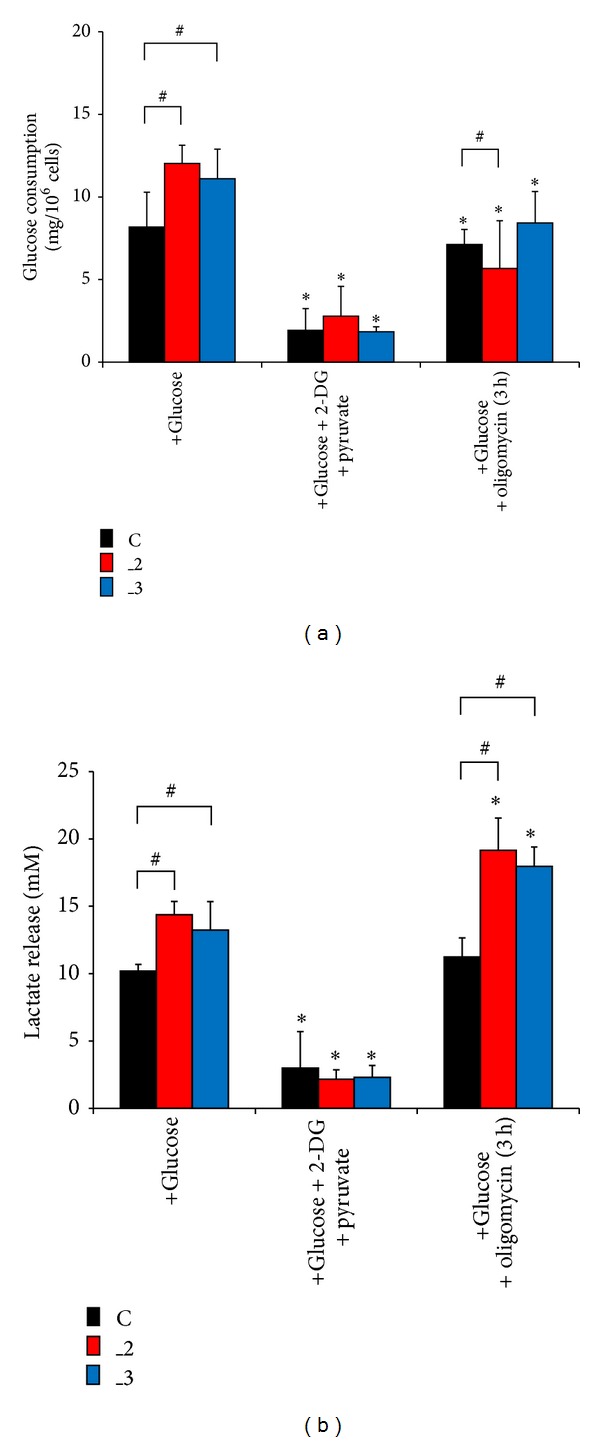
Glucose consumption and lactate release. (a) Changes in glucose concentration assessed in culture medium (+glucose) or in medium supplemented with metabolic inhibitors: 2-deoxyglucose or oligomycin. (b) Accompanied changes in lactate release determined under the same conditions. In both panels, *P < 0.05 versus nontreated cells; ^#^ *P* < 0.05 versus control cells. C: mock-transfected PC12, _2: PMCA2-reduced PC12, and _3: PMCA3-reduced PC12.

To evaluate, which energy-generating pathway was used by the cells with altered PMCA isoforms composition, we first looked into the fermentative glucose metabolism. Under standard conditions higher glucose consumption in both PMCA-reduced lines was observed (Figure 5(a)). Nearly complete loss of glucose utilization was detected when PC12 lines were cultured in the presence of 2-DG and pyruvate. Also, diminished glucose consumption was noticed after incubation with oligomycin. The lowest value was in _2 line (about 50% in relation to nontreated cells), suggesting that some portion of glucose might be preferentially metabolized to fuel OxPh.

In the presence of glucose, lactate concentration in the medium after 48 h of cells cultivation was higher by 50% in _2 and by 39% in _3 line (Figure 5(b)). Inhibition of hexokinase with 2-DG and addition of pyruvate resulted in a dramatic reduction of released lactate in all lines. Production of lactate increased in _2 and _3 lines in response to oligomycin, indicating a compensatory ATP generation through glycolysis via the Pasteur effect.

Based on the results and taking into consideration the changes in [ATP]_i_ upon 2-DG treatment, we assume that anaerobic glycolysis may be primarily responsible for supplying ATP for control and _3 lines.

### 3.5. Mitochondrial Respiration Fueled by Increased NADH Availability Meets Higher Energy Demands in PMCA2-Reduced Cells

**Figure 6:**
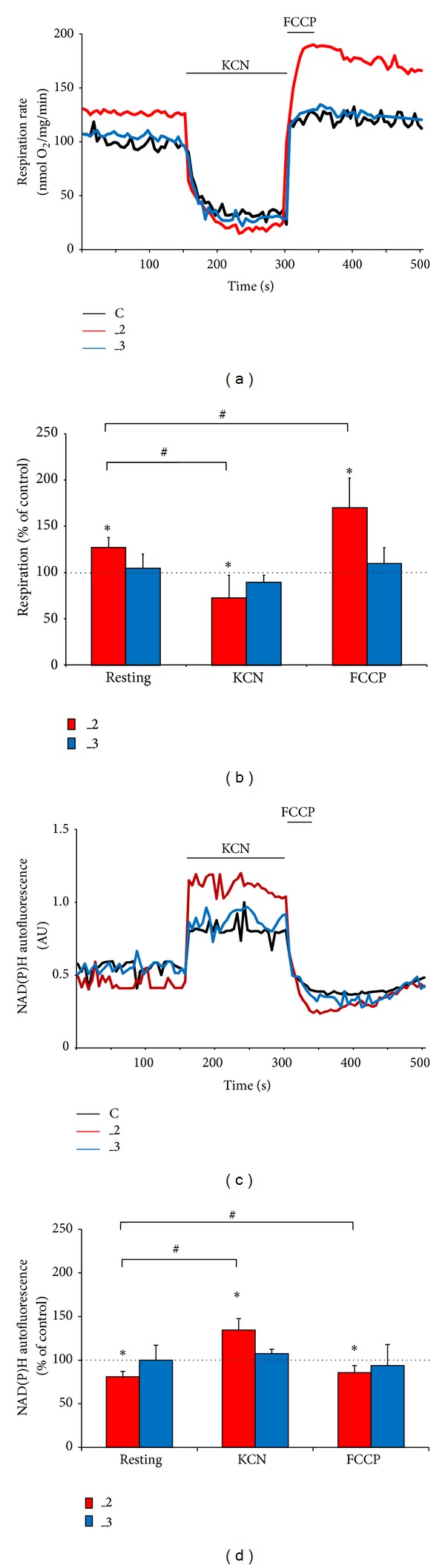
The effect of PMCAs downregulation on bioenergetic parameters. (a) The representative traces of O_2_ consumption. 2 mM KCN was used to assess minimal O_2_ consumption and 1 *μ*M FCCP to measure uncoupled respiration. (b) Quantification of respiratory rate. The respiration rate in control cells was taken as 100% (dotted line). *P < 0.05 versus control line; ^#^ *P* < 0.05 maximal or minimal O_2_ consumption versus resting. (c) Mean time course of NAD(P)H autofluorescence. 2 mM KCN and 1 *μ*M FCCP were used to obtain maximal and minimal signals, respectively. AU: arbitrary units. (d) Quantification of NAD(P)H changes. The autofluorescence level in control cells was taken as 100% and is indicated as a dotted line in the graph. *P < 0.05 versus control line; ^#^ *P* < 0.05 maximal or minimal signal versus resting. C: mock-transfected PC12, _2: PMCA2-reduced PC12, and _3: PMCA3-reduced PC12.

Based on KCN- and oligomycin-evoked changes in [ATP]_i_ we hypothesized that OxPh rather than anaerobic glycolysis is ought to provide most of the energy required for _2 cells. Indeed, _2 cells presented substantially higher basal oxygen consumption, whereas the O_2_ utilization in _3 line was comparable to control cells (Figure 6(a)). Following KCN treatment, a decline in oxygen consumption was again the most pronounced in _2 cells (about 30% below the level in control), suggesting that ATP turnover in this line might be significantly higher than in others (Figure 6(b)). Also, _2 cells presented higher maximal respiration measured in the presence of FCCP, a chemical uncoupler of electron transport and oxidative phosphorylation. Moreover, the monitoring of NAD(P)H autofluorescence revealed lower basal level of reduced NAD, greater cell response to KCN, and lowered NAD(P)H upon uncoupling the mitochondria with FCCP in _2 line (Figures 6(c), and 6(d)).

**Figure 7:**
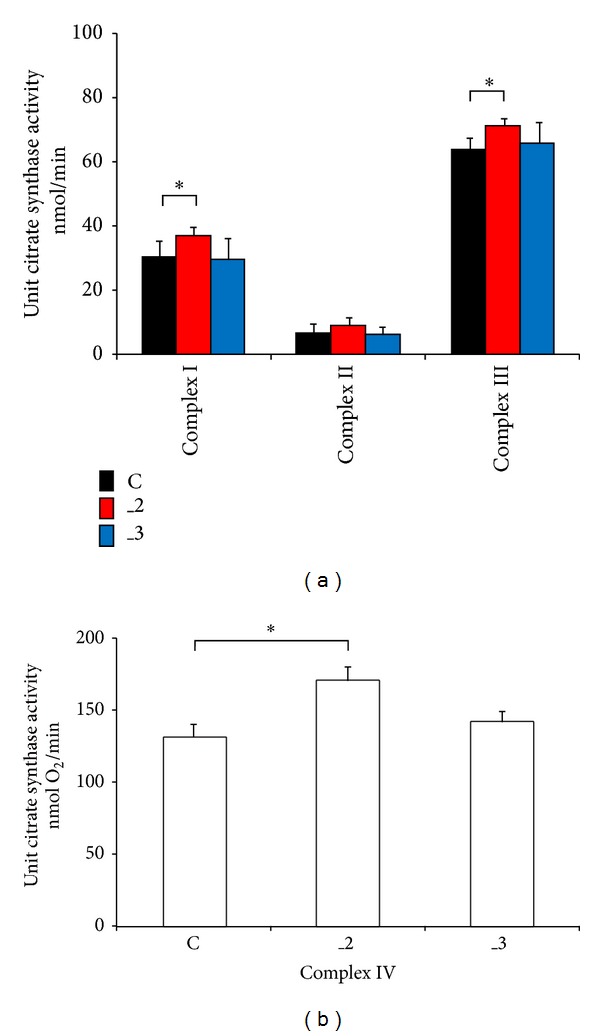
The effect of PMCAs downregulation on complexes of respiratory chain. (a) The activity of ETC complexes I–III was assessed spectrophotometrically at 37°C in mitochondrial fraction and is expressed as nmol/min after normalization to citrate synthase activity as described in Section 2. (b) Complex IV activity was determined in digitonin-permeabilized cells using an oxygraph (Anton Paar) according to the procedure described in Section 2 and is expressed as nmol O_2_/min after normalization to citrate synthase activity. The results are from *n* = 5 independent determinations. *P < 0.05 versus control cells. C: mock-transfected PC12, _2: PMCA2-reduced PC12, and _3: PMCA3-reduced PC12.

Further we assessed the activity of all four ETC complexes and normalized the results to citrate synthase activity. We observed higher activity of complexes I, III, and IV in _2 line (Figures 7(a), and 7(b)), which explained higher O_2_ utilization and together with NAD(P)H profile suggested more effective energy conversion into ATP through OxPh.

### 3.6. Oxidative Phosphorylation Is Necessary to Support Neuronal Differentiation

**Figure 8:**
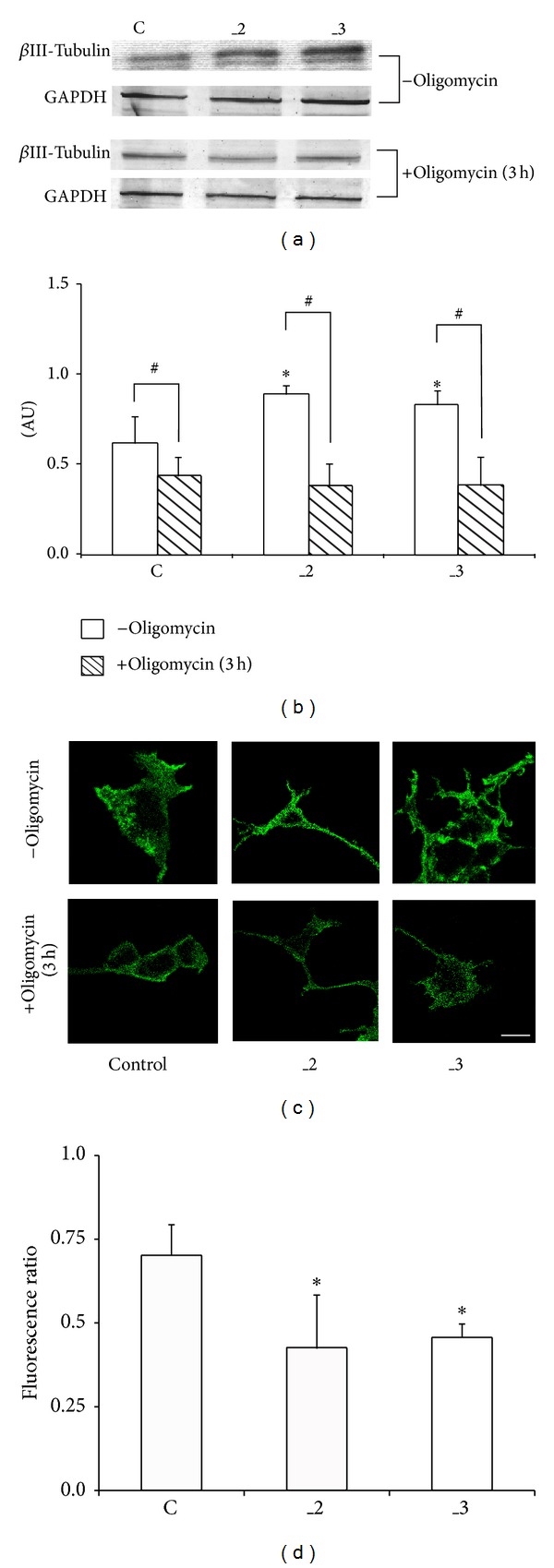
Determination of *β*III-tubulin in differentiated PC12 cell lines. (a) *β*III-Tubulin level was determined by immunoblotting in the presence or absence of oligomycin. (b) Densitometric quantification of bands intensity. The results are presented as arbitrary units (AU) obtained after normalization to endogenous GAPDH level. *P < 0.05 versus control cells; ^#^ *P* < 0.05 oligomycin treated versus nontreated cells. (c) Representative images of immunofluorescent staining of *β*III-tubulin in single cells fixed with paraformaldehyde. The images were taken using TCS SP5 confocal microscope. Scale bar 20 *μ*m. (d) Decrease in *β*III-tubulin fluorescent signal following 3 h treatment with oligomycin from randomly selected 10 cells. Fluorescence ratio was calculated as *F*_oligo_/*F*, where *F*_oligo_ is a fluorescence measured following oligomycin treatment and *F* is a corresponding fluorescence intensity in the absence of oligomycin. *P < 0.05 versus control cells. C: mock-transfected PC12, _2: PMCA2-reduced PC12, and _3: PMCA3-reduced PC12.

In view of the changes in energy generating pathways, we analyzed if OxPh is involved in differentiation process. We found that *β*III-tubulin, a protein found almost exclusively in neurons, was present at higher amounts in _2 and _3 lines in comparison to control cells. After incubation with oligomycin its level decreased to comparable values in all lines (Figures 8(a), and 8(b)). Similarly, decline in fluorescence intensity corresponding to *β*III-tubulin was visible at the single-cell level (Figures 8(c) and 8(d)). Because *β*III-tubulin is commonly considered as a neuronal marker in developmental neurobiology, our data suggest the requirement of mitochondrially generated ATP for neuronal differentiation. Moreover, PMCAs-reduced lines exhibited higher sensitivity to OxPh inhibition in terms of proper differentiation process.

## 4. Discussion

Some publications suggest that neurons contribute minimally to glucose consumption in the brain but have high rates of glucose utilization and oxidative metabolism [24, 25]. Glycolysis is thought to be crucial for neural activity [26], and the selective inhibition of hexokinase impaired the ability of neurite outgrowth in cultured adult sensory neurons [27]. Similarly, inhibition of mitochondrial ATP synthesis prevented axon formation [28] also suggesting the important contribution of OxPh to neural development. This is in agreement with studies demonstrating that neurons utilize a combination of both extracellular lactate and oxidative glucose metabolism as their energy sources [29]. Despite the ongoing debate regarding the contribution of different metabolic pathways in neurons, even less is known about the energy changes during differentiation.

In our study we used an* in vitro* model of pseudoneuronal PC12 cells with permanently downregulated PMCA2 and PMCA3 expression. We have already shown that experimental reduction in the PMCA2 or PMCA3 content in PC12 cells caused a significant drop in the efficiency of calcium extrusion [15]. The results presented here provide the evidence of a novel role of PMCA isoforms in regulation of bioenergetic pathways and mitochondrial activity and maintenance of ATP level during differentiation. In particular, profound consequences of neurospecific PMCA isoforms downregulation on energy metabolism seem to be a part of an adaptation process of both lines to altered Ca^2+^ balance. Nonetheless, we cannot exclude the contribution of compensatory upregulation of PMCA1 in both lines and PMCA4 in _3 line previously reported by us in PMCA-deficient cells [15]. In our just published paper we, however, found that manipulation of PMCA2 or PMCA3 level resulted in disruption of pH gradient across the inner mitochondrial membrane and significantly attenuated pH response during intracellular Ca^2+^ loads [30]. All the reported changes were attributed to decreased level of neurospecific PMCA isoforms and occurred even despite the induction of some compensatory mechanisms. Based on the results we concluded that PMCA2 and, to lesser extent, PMCA3 were indeed primarily responsible for driving the formation of a proton gradient and thus may exert a significant effect on cellular ATP pool. In view of these findings the potential involvement of constitutive PMCA1 and PMCA4 in bioenergetic changes reported here seems to be of minor importance.

Focusing on the differences between control and PMCAs-reduced lines, we found that ATP content in _2 line exhibited the greatest sensitivity to ETC inhibition. Higher activity of I, III, and IV respiratory chain complexes in _2 line indicates that the main source of energy for PMCA isoforms 1, 3, and 4 could be an oxidative phosphorylation. In addition, resting [Ca^2+^]_c_ in this line increased more than in _3 line, which may be crucial for activation of respiratory chain complexes. This is confirmed by ATP decrease observed in the presence of BAPTA, and also in the presence of KCN or oligomycin. However, depletion of PMCA2 which is the fastest isoform responsible for immediate clearance of increased intracellular Ca^2+^ [31–33], may lead to prolongation of Ca^2+^ signal. This, in turn, could stimulate Ca^2+^-dependent processes including TCA cycle and OxPh.

Pyruvate in the absence of glucose largely prevented ATP loss in _2 cells indicating, together with the above, greater reliance on OxPh. Several* in vitro* and* in vivo* studies demonstrated that pyruvate can protect neurons from energy deprivation and subsequent cell death [34, 35]. In contrast, only a marginal rescue effect of pyruvate observed in C and _3 lines (with unchanged PMCA2 expression) suggested the contribution of glycolysis to ATP generation. Despite different pyruvate action, increased population of necrotic cells in all lines due to glycolysis inhibition indicated that OxPh is still insufficient to fully meet energy needs, and glycolysis may be an important completive pathway.

Our data indicate PMCA2-dependent switch of cellular metabolism toward stimulation of OxPh, which could be necessary to fulfill higher ATP demands. This is supported by our findings of greater O_2_ utilization and lower NAD(P)H level, all accompanied by higher consumption of NADH by ETC due to increased activity of proton-pumping complexes. Therefore, the rate of oxidative phosphorylation when PMCA2 level is reduced may be controlled at the activity of ETC proteins which is supported by the metabolic study demonstrated in isolated mitochondria [36]. In view of the above findings, lower NAD(P)H level presumably reflects its faster oxidation. Long-time maintenance of this balance requires, however, stimulation of NADH production or its more effective transport to mitochondria. In our study, TCA cycle was found not to be a source of higher NADH synthesis, since the activity of citrate synthase and complex II of ETC remained unchanged. We cannot exclude that higher basal Ca^2+^ produced by PMCA2 downregulation may transiently activate TCA cycle dehydrogenases. This would be expected because the elevations in [Ca^2+^]_c_ are thought to increase ATP consumption by Ca^2+^ clearance mechanisms [37]. We assume that NADH transport to mitochondria rather than its overproduction may explain lower steady-state level of NAD(P)H in _2 line.

Compensation of ATP level in _2 line by pyruvate addition, when glucose was removed or 2-DG was applied, also suggests the existence of pyruvate-dependent system transferring electrons to the ETC. This phenomenon can be attributed to the stimulation of neuronal glycerol-3-phosphate shuttle, which directly transfers electrons to coenzyme Q and is regulated by Ca^2+^ and NADH/NAD^+^ level [38–40].

The correlation between cell death and ATP is still controversial. On the one hand, ATP depletion has been suggested to be a switch from apoptosis to necrosis, as ATP is required for apoptotic processes but not for necrosis [41]. Contrary, several reports demonstrated neuronal survival at very low ATP level, evidencing that energy deprivation not always correlates with neuronal death [42–44]. Our _2 and _3 lines were highly sensitive to glycolysis inhibition and both necrosis and apoptosis were detected within 3 h. Following the idea that ion homeostasis may be fuelled by ATP synthesized locally by glycolysis [45], even relatively small depletion of glycolytically derived ATP, as we observed in _2 line, may profoundly distort ion pumping and initiate death cascades.

Decreased expression of even single neurospecific PMCA isoform may impair Ca^2+^ extrusion, thereby leading to cell death due to Ca^2+^ overload. Moreover, reduction of ATP production by mitochondria can induce neuronal apoptosis or increase sensitivity to apoptotic death [46]. In line with it, cells stay alive as long as ATP is maintained at certain threshold level. Our study showed that this critical level could be sensitive to submembrane ATP consumption rate, which seems to be designated by PMCA membrane composition. Based on this we hypothesize that presence of PMCA2 and PMCA3 during neuronal differentiation may confer a survival advantage.

It is well known that calcium ions upregulate signaling pathways engaged in cell differentiation [11, 47, 48]. Thus, more intensive ATP synthesis by OxPh could explain more intense differentiation of _2 line. Control and _3 lines, possessing normal level of PMCA2, rely mainly on ATP produced by glycolysis suggesting the predominance of this energetic pathway in energy provision for this isoform. Taking both into account, the plasma membrane location of calcium pump, as well as the presence in close proximity of glycolytic enzymes, seems to be suitable for fast ATP harvesting in excitable cells. Availability of ATP necessary for quick response to increasing Ca^2+^ may be therefore crucial for ion homeostasis maintenance.

In summary, our findings demonstrate that changes in ATP pool during differentiation are tightly coupled to Ca^2+^ signaling. Disruption in Ca^2+^ balance, as a result of PMCA2 or PMCA3 downregulation, may in turn profoundly affect cellular bioenergetic processes. Depending whether prolonged Ca^2+^ signal was originated from PMCA2 or PMCA3 reduced clearing potency, cells upregulate different energy-generating pathways to keep ATP level above the threshold necessary for survival. In our experimental model, this adaptive response may represent a feedback mechanism of ATP supply to maintain cellular ion homeostasis. This would be particularly beneficial when PMCA2 level is reduced and cells are exposed to protracting periods of mild calcium elevations. These relatively small variations in [Ca^2+^]_c_ are, however, able to modify mitochondrial metabolism suggesting a tight relationship between PMCA2 and ATP production. Because even small changes in cellular energy state may initiate cellular pathology and death, fast reacting PMCA2 and PMCA3 isoforms may be considered as cellular bodyguards protecting against Ca^2+^ overload and subsequent mitochondrial failure.

## Figures and Tables

**Table 1:** Relationship between cytosolic Ca^2+^ and ATP level. Changes in total cellular ATP level upon BAPTA treatment are expressed as % change in relation to nontreated line, in which ATP level in particular line was taken as 100%. The values of ATP concentration in steady state noninhibitory conditions are as follows: 3.66 ± 0.32, 4.72 ± 0.39, and 3.43 ± 0.66 nmol/mg for C, _2, and _3 lines, respectively, and these values are also presented in Figure 2 (conditions “+glucose”).

	C	_2	_3
[Ca^2+^]_c_ [nM]	95 ± 8	149 ± 17∗	130 ± 11∗
[Ca^2+^]_c_ + BAPTA [nM]	90 ± 14	100 ± 11^#^	96 ± 15^#^
ATP + BAPTA [%]	103 ± 10	80 ± 5^#^	94 ± 8

*P < 0.05 versus control untreated cells; ^#^ *P* < 0.05 treated versus untreated cells. C: mock-transfected PC12, _2: PMCA2-reduced PC12, and _3: PMCA3-reduced PC12.
